# Adult Distal Duodenal Obstruction: A Diagnostic and Therapeutic Challenge

**DOI:** 10.7759/cureus.24095

**Published:** 2022-04-13

**Authors:** Joel Thomas, Karen Abraham, Dixon Osilli, Samrat Mukherjee

**Affiliations:** 1 Radiology, Barking, Havering and Redbridge University Hospitals National Health Service (NHS) Trust, Romford, GBR; 2 General Surgery, Barking, Havering and Redbridge University Hospitals National Health Service (NHS) Trust, Romford, GBR

**Keywords:** carcinoma, gastric outlet obstruction, duodenal obstruction, obstruction, duodenum

## Abstract

Distal duodenal obstruction (DDO) can be succinctly defined as features of gastric outlet obstruction with bilious vomiting and radiological or endoscopic evidence of post-bulbar obstruction. Obstructions of the third (D3) and fourth (D4) parts of the duodenum are rare and present significant diagnostic and surgical challenges, particularly when the cause is malignant. In the following three case reports, we discuss three distinct aetiologies of this rare syndrome and highlight important considerations surrounding the early investigation and management of these individuals.

The first patient is a 60-year-old lady with primary duodenal adenocarcinoma resulting in malignant stricture at D4. She underwent segmental resection of the D4 tumour with a duodeno-jejunal anastomosis. The second patient is a 17-year-old boy with superior mesenteric artery (SMA) syndrome, who was treated conservatively. The last patient is a 71-year-old lady with a caecal carcinoma invading the retroperitoneal structures and D3. The patient underwent a palliative laparoscopic gastro-jejunostomy.

Although infrequently encountered in clinical practice, the individual burden of a missed or late diagnosis of DDO, malignant or otherwise, can be disastrous. This case series illustrates the varied presentation of DDO and discusses current principles of investigation and management.

## Introduction

The duodenum is primarily a retroperitoneal organ that begins at the pylorus and ends at the ligament of Treitz. It measures approximately 20 cm and consists of four segments. The transversely oriented first portion (D1) begins at the pylorus and ends at the common bile duct superiorly and the gastroduodenal artery inferiorly. The second portion (D2) runs inferiorly to the ampulla of Vater. The third portion (D3) runs transversely to the level of the superior mesenteric artery (SMA) and vein, and the fourth portion (D4) extends to the point where the duodenum emerges from the retroperitoneum to join the jejunum at the left border of the second lumbar vertebra.

Various pathologies in the distal duodenum (both intrinsic and extrinsic) can give rise to the features of duodenal obstruction [1]. Their aetiologies are diverse and vary significantly depending on the patient’s age [2]: duodenal atresia, duodenal webs, and annular pancreas are usually seen in infancy while peptic ulceration, inflammatory strictures, and malignancy (primary or secondary) are more common in the adult population. The vague symptomatology in the early stages is compounded by the fact that D3/D4 is not reached by conventional endoscopy. In one case series of 18 patients, 83% of initial endoscopies were negative - resulting in late presentation and high rates of misdiagnosis [3].

We present three case reports of patients with distal duodenal obstruction (DDO), all differing significantly in aetiology and background, and discuss our approaches to investigation and treatment.

## Case presentation

Case 1

A 61-year-old lady, previously fit and well with no past medical history, was admitted as an emergency following a collapse at home. This was on the background of a six-month history of recurrent vomiting and weight loss (>7kg).

**Figure 1:**
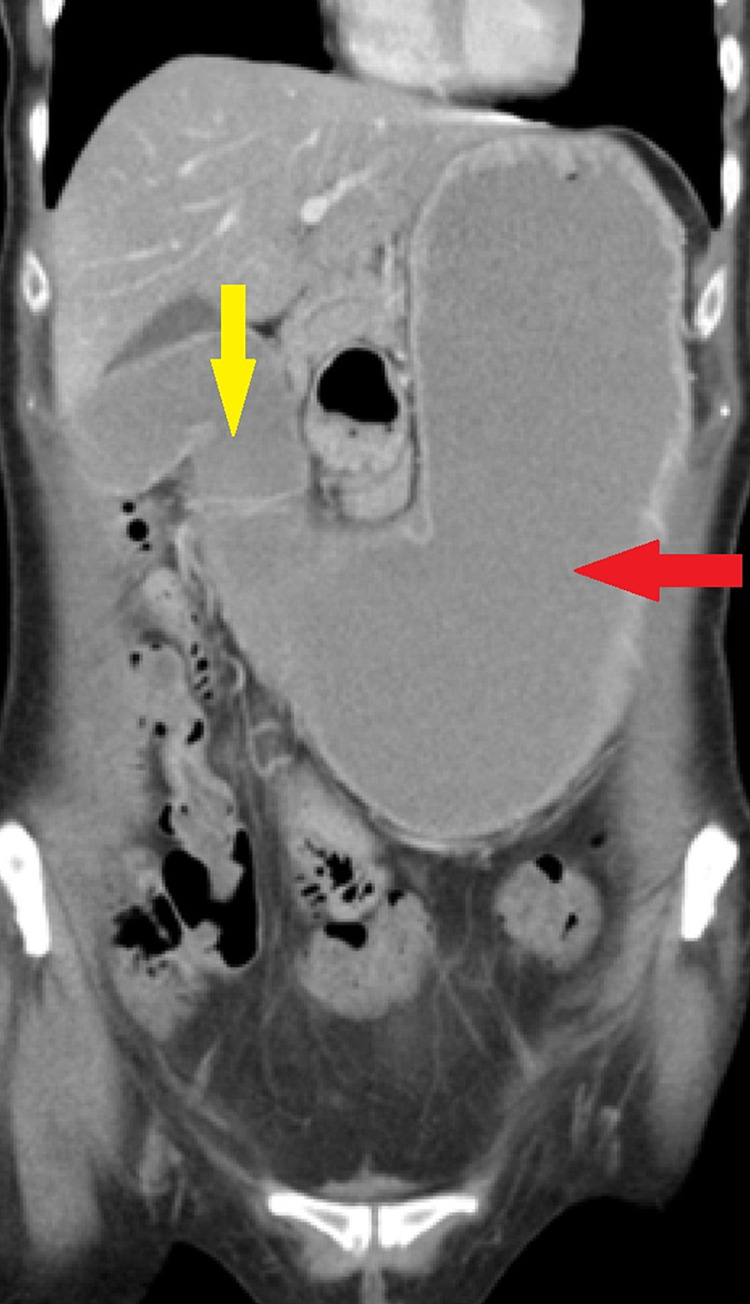
CT scan showing grossly distended stomach (red arrow) with thickening of the pylorus (yellow arrow) and distension of the proximal duodenum.

Over the preceding six months, she had been admitted multiple times under gastroenterology with symptoms of vomiting. An initial gastroscopy in this period revealed hiatus hernia, gastritis and oesophagitis (resulting in proton pump inhibitor [PPI] therapy), while an abdominal computed tomography (CT) scan had shown a distended stomach and duodenum with mural thickening of the pylorus (Figure 1).

On initial review, this lady appeared emaciated and dehydrated but with a soft non-tender abdomen. Blood tests were only significant in slightly raised urea and creatinine. She was started on IV fluids with a plan to obtain a repeat abdominal CT and gastroscopy. The repeat abdominal CT scan showed a markedly distended stomach with obstruction at D3/D4. Push enteroscopy revealed a duodenal tumour at D4 with malignant stricture and complete obstruction - biopsies confirmed a moderately differentiated invasive adenocarcinoma of the intestinal type.

The patient underwent an open resection of the D4 tumour with duodeno-jejunal anastomosis and good lymph node status. She completed her adjuvant chemotherapy in May 2019 and is currently well and asymptomatic with a performance status of 0.

Case 2

**Figure 2:**
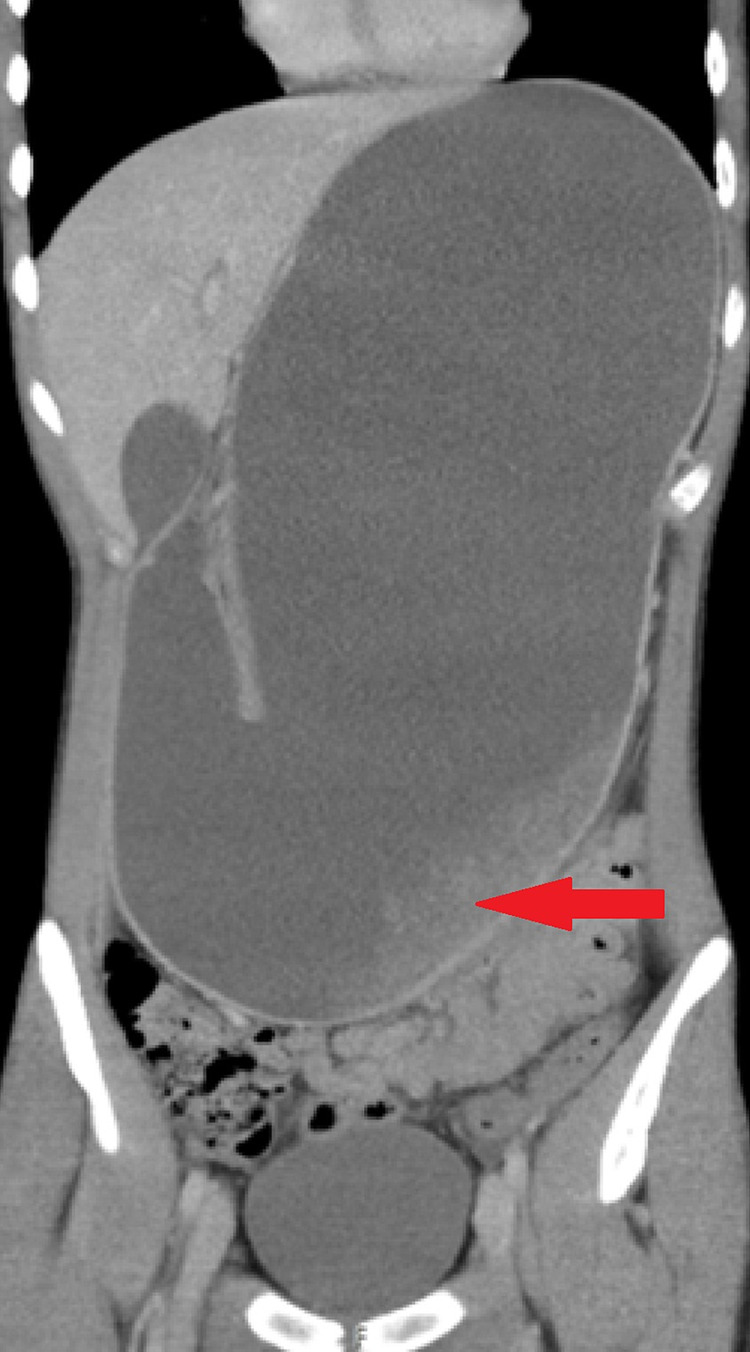
CT scan showing grossly distended stomach. The duodenum is not visible. Hazy intra-gastric opacification (red arrow) is likely to be food residue.

A 17-year-old boy presented to Accident & Emergency with vomiting and a distended abdomen, while his blood tests showed deranged renal function with a widespread elevation of non-specific markers (indicative of dehydration). CT showed a grossly dilated stomach with a cut-off at D3 (Figure 2) and an urgent MRI revealed the cause as SMA syndrome.

In coordination with our dietitian team, he was treated conservatively with parenteral nutrition and a gradual increase in diet over four weeks of inpatient stay. His symptoms resolved and at 14 months follow-up he has regained weight and is eating and drinking normally with no further episodes of illness.

Case 3

A 71-year-old lady presented with a five-week history of abdominal pain but no vomiting or weight loss. Her past medical history included type II diabetes, hypertension and hypercholesterolaemia. Physical examination was unremarkable and her blood tests revealed only mild normocytic anaemia.

**Figure 3:**
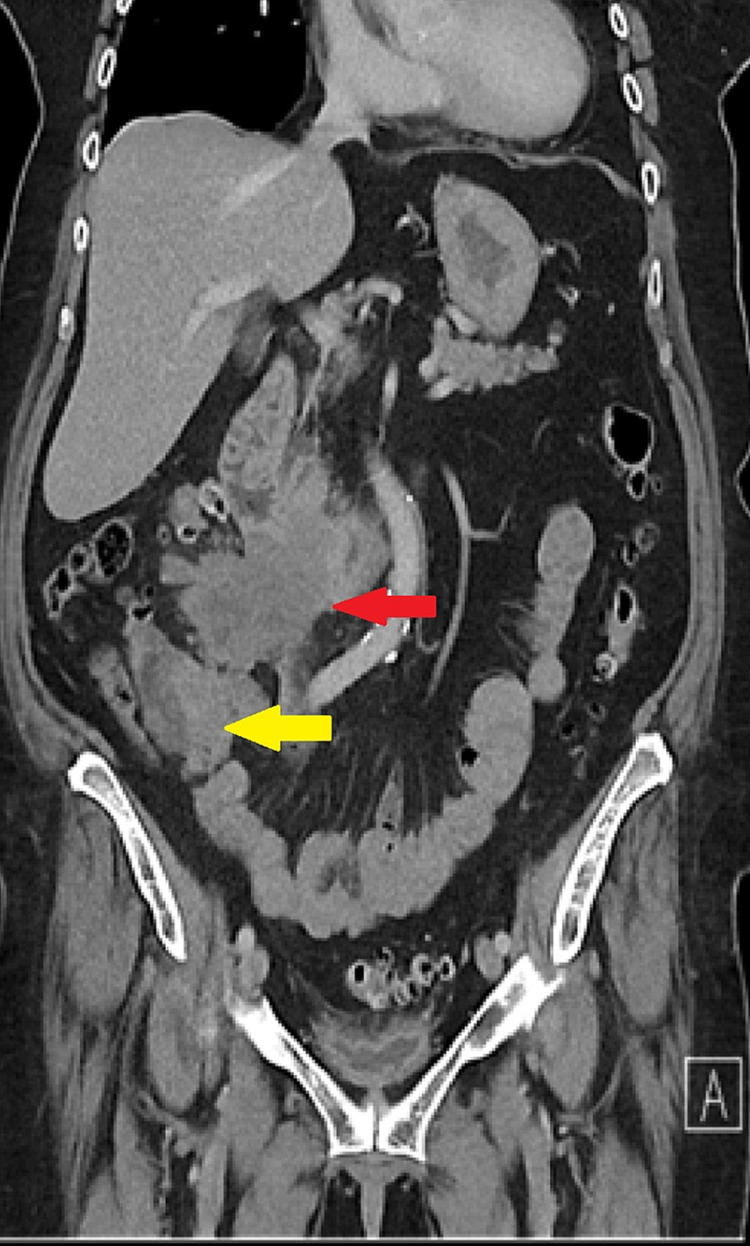
CT scan showing thickened D3 segment leading to a large irregular mass (red arrow). This is closely associated with the primary caecal mass (yellow arrow).

An abdominal CT scan revealed invasion of the retroperitoneum and D3 segment by a caecal malignancy (Figure 3), with a subsequent gastroscopy showing a stricture beyond the ampulla of Vater. A colonoscopy confirmed a caecal tumour. Biopsies from both endoscopies were consistent with a primary caecal adenocarcinoma metastasising to the duodenum.

Due to the location and advanced stage of the tumour, the patient was considered unfit for radical intervention. She underwent a palliative laparoscopic gastro-jejunostomy to enable her to eat. Her symptoms significantly reduced and she passed away in hospice six months after surgery.

## Discussion

Clinical presentation

Patients with DDO - regardless of pathology - present with diverse symptoms including early satiety, intermittent vomiting, epigastric pain/discomfort, abdominal distension, and weight loss. These symptoms overlap with the main differential of gastric outlet obstruction (GOO) - though bilious vomiting, dark green in appearance, is an indicator of obstruction beyond the pylorus. Nevertheless, it is still quite challenging to differentiate between DDO and GOO based on symptoms alone.

In one series of 49 patients, the most common clinical features were epigastric pain (94%), vomiting (92%), and weight loss (63%). The majority of patients (78%) had the malignant disease. In another cohort of 30 patients with benign causes of GOO, the most common presenting features were early satiety (53%) and bloating (50%) [4].

Like history, the physical examination usually yields non-specific findings which can lead to delays in diagnosis and the development of fatal complications [5].

There are many potential causes of DDO in adults, most commonly including duodenal neoplasms (benign and malignant; primary or secondary), pancreatic pathology (severe acute or chronic pancreatitis, pancreatic pseudocysts, and pancreatic neoplasms), hepatobiliary pathology (neoplasms, Bouveret syndrome) post-bulbar peptic ulcer disease, Crohn’s disease, SMA syndrome, and retroperitoneal disease.,

The diagnosis may be suspected based upon the clinical features outlined above and is confirmed by radiologic evaluation and/or endoscopy.

Investigations

Laboratory tests may be normal or non-specifically abnormal. As with any other cause of recurrent vomiting, patients may have electrolyte abnormalities including hypokalemia or a hypochloremic metabolic alkalosis. Anaemia may be seen in patients with peptic ulcer disease, primary or metastatic malignant disease, or large gastric polyps, but in the context of a clinical picture suggesting DDO it most often signifies malignancy. Serum tumour markers such as CA 19-9 and CEA may also indicate a malignant process but elevated levels can be found in other diseases (both benign and malignant) of the gastrointestinal tract.

Plain Films

Plain films of the abdomen may reveal an enlarged gastric bubble and a dilated proximal duodenum. A paucity of air in the small bowel is often noted. The plain film can also sometimes suggest an underlying cause such as a gallstone or pancreatitis. A calcified mass in the right upper quadrant is seen in up to 25% of patients with Bouveret syndrome [6], whereas pancreatic calcifications are suggestive of chronic pancreatitis.

Contrast Studies

Contrast studies have now been largely replaced by CT and endoscopy. However, they may be useful if a partial obstruction is suspected. 

CT Scan

An abdominal computed tomography (CT) scan may reveal gastric distension, along with retained material within the gastric lumen and an associated air-fluid level. CT will often also suggest the specific cause of the obstruction.

Endoscopy

In most cases, upper endoscopy is the modality of choice to identify the specific cause and establish a tissue diagnosis. It can also permit therapeutic procedures.

Endoscopic biopsies often allow for confirmation or exclusion of a malignant cause of DDO. However, routine biopsy techniques can have poor sensitivity, particularly if the tumour is extraluminal or does not involve the mucosa [7]. Thus, patients with negative initial biopsies who are felt to be at increased risk for a malignancy should undergo additional testing. Patients should be considered at increased risk if they are older than 50 years of age and do not have a history of peptic ulcer disease, Crohn's disease, or do have a family history of gastro-duodenal cancer [8]. Additional evaluation may include multiple tunnelled endoscopic biopsies, endoscopic ultrasound (EUS), or full-thickness surgical biopsies. A CT scan should be performed if extrinsic compression is suspected.

Endoscopic biopsies have a poor yield for the diagnosis of gastroduodenal tuberculosis; surgical specimens are usually required [9]. Endoscopy in the setting of Bouveret syndrome reveals the offending stone in only 70% of cases, likely due to mucosa overlying the embedded stone [10].

The management of DDO is guided by the underlying cause of the obstruction, although all patients will benefit from good supportive care and symptom control. Symptomatic relief should be offered to all patients, including PPIs and pharmacological management for nausea and vomiting. Most patients will require dietitian input during the course of their illness, and the use of modified eating habits, oral supplements, or intravenous nutrition may be considered. All of these conservative approaches can significantly improve the patient’s quality of life as well as strengthen their physiological state and thereby increase their suitability for invasive interventions.

For distal duodenal carcinomas, the choice in surgical approach is between segmentectomy and the more radical pancreaticoduodenectomy, with the aim to offer the patient the highest possible survival chances. Although some surgeons prefer pancreaticoduodenectomy as a way of minimising the risk of retained malignant tissue, this comes at the price of significantly higher post-operative morbidity and mortality [11]. More recent research is now yielding highly useful insights for operating teams. It was found that the more limited segmentectomy appears to offer statistically equivalent long-term survival rates with considerably less morbidity and that specific pre-operative factors including tumour, node and metastasis (TNM) stage, tumour grade on histology, and use of radiotherapy are all more reliable predictors of patient outcomes than the type of resection [12]. This may help guide clinician and patient choices between more or less radical surgical approaches.

Available evidence from randomised controlled trials (RCTs) suggests that despite comparable benefits and complication rates, stents are associated with many positive metrics compared to gastro-jejunostomy, including reduced hospital stays, lower post-operative mortality, and faster relief of symptoms [13]. Recurrence of obstructive symptoms does appear to be significantly more likely after stent placement [14], which may encourage stronger consideration of gastro-jejuostomy in patients with longer prognoses to avoid the need for re-intervention.

## Conclusions

Obstruction of the distal duodenum produces vague and confounding symptoms, and is often resistant to early attempts at diagnosis. A high degree of suspicion should lead to a further endoscopic investigation, including push enteroscopy and EUS. Given the underlying anatomical and pathological complexities, the choice of treatment option is difficult and, if possible, should only be undertaken by a suitable multidisciplinary team (MDT).
